# Overexpression of HLH4 Inhibits Cell Elongation and Anthocyanin Biosynthesis in *Arabidopsis thaliana*

**DOI:** 10.3390/cells11071087

**Published:** 2022-03-24

**Authors:** Quancan Hou, Wei Zhao, Lu Lu, Linlin Wang, Tianye Zhang, Binbin Hu, Tingwei Yan, Yuchen Qi, Fan Zhang, Nan Chao, Dorothea Bartels, Xiangyuan Wan

**Affiliations:** 1 Zhongzhi International Institute of Agricultural Biosciences, Shunde Graduate School, Research Center of Biology and Agriculture, University of Science and Technology Beijing (USTB), Beijing 100024, China; houquancan@ustb.edu.cn (Q.H.); b20190396@xs.ustb.edu.cn (W.Z.); g20198927@xs.ustb.edu.cn (L.L.); s20200896@xs.ustb.edu.cn (L.W.); s20200906@xs.ustb.edu.cn (T.Z.); b20190395@xs.ustb.edu.cn (T.Y.); m202110892@xs.ustb.edu.cn (Y.Q.); b20200418@xs.ustb.edu.cn (F.Z.); 2 Beijing Engineering Laboratory of Main Crop Bio-Tech Breeding, Beijing International Science and Technology Cooperation Base of Bio-Tech Breeding, Beijing Solidwill Sci-Tech Co., Ltd., Beijing 100192, China; 3 Institute of Molecular Physiology and Biotechnology of Plants, University of Bonn, Kirschallee 1, 53315 Bonn, Germany; 4 Institute of Botany, Chinese Academy of Sciences (CAS), Beijing 100093, China; hubinbin@ibcas.ac.cn; 5 College of Biotechnology, Jiangsu University of Science and Technology, Zhenjiang 212018, China; 201900000066@just.edu.cn

**Keywords:** anthocyanin biosynthesis, cell elongation, bHLH transcription factor, HLH4, HLH/bHLH triantagonistic system

## Abstract

In plants, many basic helix-loop-helix (bHLH) transcription factors are involved in controlling cell elongation. Three bHLH proteins, PACLOBTRAZOL RESISTANCE1 (PRE1), Cryptochrome Interacting Basic Helix-loop-helix 5 (CIB5), and Arabidopsis ILI1 binding bHLH1 (IBH1) form a triantagonistic system that antagonistically regulates cell elongation in a competitive manner. In this study, we identified a new player, HLH4, related to IBH1, that negatively regulates cell elongation in *Arabidopsis thaliana*. Overexpression of HLH4 causes dwarf and dark green phenotypes and results in the downregulation of many key regulatory and enzymatic genes that participate in the anthocyanin biosynthetic pathway. HLH4 interacts with CIB5 and PRE1. By interacting with CIB5, HLH4 interferes with the activity of CIB5, and thus inhibiting the transcription of cell elongation-related genes regulated by CIB5, including *EXPANSINS8* and *11* (*EXP8* and *EXP11*) and *indole-3-acetic acid* 7 and 17 (*IAA7* and *IAA17*). The interference of HLH4 on CIB5 is counteracted by PRE1, in which these bHLH proteins form a new tri-antagonistic system.

## 1. Introduction

Cell elongation is essential for plant morphogenesis, growth, and response to environmental changes. Plant cell elongation is regulated by endogenous phytohormones such as auxin, brassinosteroids (BRs), and gibberellins (GAs) [1]. In addition, environmental factors such as light regulate plant cell elongation. For example, in the presence of light plants undergo photomorphogenesis, develop shortened hypocotyls and opened cotyledons, and initiate chlorophyll and anthocyanin production [2]. On the contrary, when seeds germinate in the dark seedling hypocotyls are elongated and cotyledons and apical hooks are closed, which is known as skotomorphogenesis [3]. Far-red light and blue light modulate plant cell elongation in a specific manner [4,5]. This light-regulated cell elongation, known as the shade avoidance response, enables plants to obtain sufficient light for photosynthesis. Hormonal and environmental signals interact and regulate cell elongation in a complex way. Studies have demonstrated the integration of the auxin, BR, GA, light, and temperature pathways through interactions of their target transcription regulators [6,7,8,9].

Various transcription regulators, including the basic helix-loop-helix (bHLH) transcription factors, have been shown to control cell elongation. In *Arabidopsis thaliana*, the Phytochrome-Interacting Factors (PIF) PIF4 and PIF5 positively regulate shade-induced elongation, as *pif4* and *pif5* display reduced hypocotyl elongation and the expression of shade-responsive genes is downregulated [10]. Long Hypocotyl in Far-Red1 (HFR1) and Phytochrome Rapidly Regulated1 (PAR1), two atypical non-DNA binding bHLH proteins, form heterodimers with PIF4 to interfere with the DNA binding activity of PIF4. Thus, HFR1 and PAR1 play negative roles in shade avoidance responses [11]. Paclobutrazol Resistance (PRE) subfamily proteins, including PRE1, PRE3, and PRE6, are atypical non-DNA binding bHLH proteins which positively regulate cell elongation in plant response to GA, BR, and light signaling [12,13,14]. PRE1 suppresses the dwarf phenotype of the GA biosynthesis-defective mutant *ga2-201*, indicating that PRE1 is involved in GA-regulated cell elongation [13]. Unlike *Arabidopsis*, ectopic expression of the rice PRE1 ortholog Increased Leaf Inclination1 (ILI1) in rice and *Arabidopsis* caused a BR-sensitive phenotype [15]. BR signaling activates the Brassinazole-Resistant1 (BZR1) and BZR2/BES1 transcription factors, which further regulate the expression of *PRE1* and genes related to cell wall biosynthesis and modification, including *Xyloglucan endotransglucosylase* (*XTH*) and *EXPANSIN* [16,17,18]. In rice, BZR1 directly represses the expression of an ILI1-binding bHLH1 (IBH1) protein which acts as a negative regulator of cell elongation; ectopic expression of the *Arabidopsis* orthologue of IBH1 induces a dwarf phenotype [15]. However, ectopic expression of PRE1, PRE3/ATBS1, and PRE6/KDR suppressed the dwarf or short hypocotyl phenotypes induced by the ectopic expression of IBH1, ATBS1-Interacting Factors (ARFs), and HFR1, respectively. Several bHLH transcriptional activators, Activator of Cell Elongations (ACEs) and Cryptochrome Interacting Basic Helix-loop-helix (CIB) CIB1 and CIB5 were identified as positively regulating cell elongation in response to GA and BR signaling [19,20]. IBH1, PRE1, and ACEs or CIB5 form a tri-antagonistic HLH/bHLH system in which ACE1 and CIB5 activate the expression of genes involved in cell elongation, IBH1 negatively regulates cell elongation by forming heterodimers with ACEs or CIB5 to suppress their DNA binding activity, and PRE1 interacts with IBH1 to interfere with its interaction with ACEs or CIB5 to positively regulate cell elongation [20]. Similarly, CIB1 and PAR1 form an antagonistic HLH/bHLH system in which expression of *CIB1* is regulated by another antagonistic HLH/bHLH system composed of HFR1, PAR1, and PIF4 [19].

As described above, light is a regulator of cell elongation. Excess light causes DNA damage, generates excess reactive oxygen species, and impairs photosynthesis [21]. Plants accumulate “sunscreen” flavonoids, a kind of low molecular weight phenolic compounds that includes anthocyanins, proanthocyanidins, and flavonol, to help limit damage induced by excess light [22]. In *Arabidopsis*, biosynthesis of anthocyanin is initiated from the flavonoid synthetic pathway and involves multiple enzymes [23]. Early steps for flavonoid biosynthesis are catalyzed by enzymes including chalcone synthase (CHS), flavonol 3-hydroxylase (F3H), and chalcone isomerase (CHI), and later steps for anthocyanin biosynthesis require leucoanthocyanidin dioxygenase (LDOX), dihydroflavonol-4-reductase (DFR), and UDP-glucose:flavonoid-3-O-glycosyl-transferase (UF3GT) [24]. MYB, bHLH, and WD40-repeat transcription factors including MYB75, MYB90, MYB113, TRANSPARENT TESTA 8 (TT8), and TRANSPARENT TESTA GLABRA 1 (TTG1) form a protein complex (MBW) that regulates the expression of the enzyme-encoding genes [25]. Several factors that are involved in both anthocyanin biosynthesis and hypocotyl elongation were identified from apple. A B-BOX protein, MdBBX37, interacts with two key positive regulators of anthocyanin biosynthesis, MdMYB1 and MdMYB9, and inhibits DNA binding of their target genes, thereby negatively regulating anthocyanin biosynthesis. MdBBX37 directly regulates *MdHY5* and suppresses its expression, thus relieving MdHY5-mediated hypocotyl inhibition [26]. Auxin regulates the elongation and division of plant cells. Auxin signaling activates auxin response factor (ARF) family transcription factors by degrading the AUX/IAA family proteins, which are inhibitors of ARFs [27]. Overexpression of *MdIAA26* in apple calli or *Arabidopsis* promotes the accumulation of anthocyanin and upregulation of anthocyanin biosynthesis-related genes [28]. Overexpression of *MdIAA121* weakened the inhibition of MdARF13 on anthocyanin biosynthesis in apple, leading to anthocyanin accumulation [29]. These findings indicate that auxin regulates anthocyanin biosynthesis via the Aux/IAA–ARF signaling cascade.

In this study, we identified a new player, HLH4 which is related to IBH1 and negatively regulates cell elongation in *A. thaliana*. HLH4 interacts with other bHLH proteins, including CIB5, which are involved in the regulation of cell elongation. By interacting with these regulators, HLH4 interferes with their activity, leading to cell elongation suppression. We show that the interference of HLH4 is counteracted by the other type of atypical bHLH proteins, PREs; these bHLH proteins form a new tri-antagonistic system. Ectopic overexpression of *HLH4* results in the downregulation of many key regulatory and enzymatic genes that participate in the anthocyanin biosynthesis pathway, suggesting HLH4 may have a regulatory role in anthocyanin biosynthesis in *A. thaliana*.

## 2. Materials and Methods

### 2.1. Plant Growth Conditions and Stress Treatments

Seeds of wild-type *A. thaliana* ecotype Col-0 (Columbia-0) were originally obtained from the Nottingham Arabidopsis Stock Centre, UK. Seeds were germinated and plants were grown in potting soil under conditions with a day/night (22 °C/18 °C) cycle of 8/16 h in white light of around 150 µmol m^−2^ s^−1^. All experiments were performed with approximately four-week-old soil-grown plants if not otherwise stated. Light stress was provided by 1000 µmol m^−2^ s^−1^ for one week. All treated plant material was used immediately for experiments or frozen in liquid nitrogen and stored at −80 °C for further analyses.

### 2.2. Chlorophyll and Anthocyanin Extraction

Leaf tissue (3–4 mg) was collected using a hole puncher, and the weight was recorded. The leaf disk was then transferred into a tube with 800 µL DMF (*N*,*N*-Dimethylformamide) and incubated for 4 h in the dark. The absorbance of each tube was measured at 647 nm and 664 nm using a spectrophotometer. Total chlorophyll (µg Chl/mg fresh weight) was calculated by the formula 0.8 × ((17.67 × A664) + (7.17 × A647))/fresh weight.

Anthocyanin was extracted following a protocol described previously [30]. Specifically, leaf disks were weighed and ground in liquid nitrogen. The powder was incubated in 300 µL of methanol acidified with 1% HCl overnight. Then, 200 µL of distilled water and 500 µL of chloroform were added to separate anthocyanins from chlorophylls, 400 µL aqueous phase was transferred to a new tube, and in order to bring the volume up to 800 µL, 400 µL of 60% Methanol 1% HCl was added. The absorbance of each sample was measured at 530 nm and 657 nm using a spectrophotometer. The formula “(A530–A657) × 1000/fresh weight” was used for anthocyanin content calculation.

### 2.3. Propidium Iodide Staining

Seedlings of *A. thaliana* were stained with 10 μg/mL PI (Sigma, Shanghai, China) for 2 min. Excess staining was removed by rinsing seedlings in water. Images were captured using a Leica SP8 with excitation at 514 nm and emission at 617 nm.

### 2.4. GUS Histochemical Staining

Freshly collected tissue samples of *pHLH4-GUS* transgenic *A. thaliana* plants were stained in 0.1 M sodium phosphate buffer (pH 7.0) containing 0.5 mM K_3_Fe(CN)_6_, 1 mM K_4_Fe(CN)_6_, 0.05% Triton X-100, and 0.1% X-gluc for 4 h and then incubated in 70% ethanol for 30 min three times. 

### 2.5. Subcellular Localization Analysis

The HLH4 coding sequences were amplified from genomic DNA and fused in-frame to the 5′ of GFP coding sequence, resulting in the HLH4-GFP. The HLH4-GFP fragment was then cloned into the vector pUC19-35S-FLAG-RBS (GenBank accession no. DQ077692) via infusion method to substitute the FLAG coding sequence. The final construct harboring the 35S-HLH4-GFP was transformed into *A. thaliana* protoplasts. GFP and chlorophyll fluorescence from protoplasts were visualized under a confocal microscope (TCS-SP8, Leica). Primers used for the plasmid construction are listed in Table S4.

### 2.6. Quantitative Real-Time PCR (qRT-PCR) Analysis

Total RNA was isolated using the TRIzol reagent (Invitrogen, Carlsbad, CA, USA) following the manufacturer’s instructions. Total RNA (1.5 μg) was used to synthesize the first-strand cDNA using the 5×All-In-One RT MasterMix (abm, Richmond, Canada). RT-PCR was performed using 1 μL cDNA as templates with a program of 95 °C for 3 min, 25 cycles of 95 °C for 10 s, 60 °C for 25 s, and 72 °C for 30 s. qRT-PCR was performed on a QuantStudio5 Real-Time PCR System (Thermo Fisher, Carlsbad, CA, USA) in triplicate using TB Green Premix Ex Taq (Takara, Kyoto, Japan) according to the manufacturer’s instruction. qRT-PCR was run with a program of 95 °C for 10 min, 40 cycles of 95 °C for 10 s, 60 °C for 25 s, and 72 °C for 20 s. *Act2* (*At3g18780*) was used as an internal control. All the primers used for RT-PCR and qRT-PCR are listed in Table S4. Data were analyzed by the 2^−ΔΔCt^ method and quantitative results were given as means ± SD.

### 2.7. Transient Dual-Luciferase Assay

For the transcriptional activity analysis, the coding regions of *CIB5* and *HLH4* were amplified and cloned into the *Bam*HI/*Sal*I-digested pRT-BD vector (35S-Ω-GAL4 DBD-NOS) or *Eco*RI-digested BD-VP16 vector by homologous recombination. These constructs were used for expressing effectors. The plasmids of 5× GAL4-TATA:LUC and proAtUbiquitin3:REN were used as a reporter gene and internal control, respectively (Wei et al., 2009). For promoter transactivation assay, the promoter regions of investigated genes were amplified from *A. thaliana* genomic DNA and cloned into the *Kpn*I/*Bam*HI-digested pEASY-LUC vector by homologous recombination. The constructed plasmids were used as reporters. To generate effector plasmids, the coding regions of investigated genes were cloned into the *Sac*I/*Xba*I-digested pRT-BD vector (replacing the GAL4 DBD region). The proAtUbiquitin3:REN plasmid was used as an internal control. Relative luciferase activity (LUC/REN) was determined in *A. thaliana* protoplast transient expression systems as described previously [31]. Primers used for the plasmid construction are listed in Table S4.

### 2.8. Electrophoretic Mobility Shift Assay (EMSA)

The EMSA assay was performed using a DIG High Prime DNA Labeling and Detection Starter Kit II (Roche) according to the manufacturer’s instruction. Briefly, the *HLH4* coding sequence was amplified from *A. thaliana* genomic DNA and cloned in-frame into the pET28a vector containing N-terminal His tags. The plasmid was transformed into *Escherichia coli* BL21 (DE3) cells and purified using Ni-charged resin (Cat#1560131, BioRad, Hercules, CA, USA) following the manufacturer’s instructions. The purified protein was desalted using PD-10 desalting columns (GE Healthcare, Little Chalfont, UK) and used for EMSA.

### 2.9. Yeast Two-Hybrid (Y2H) Assay

For yeast two-hybrid library screening, the coding sequence of *HLH4* was cloned into the pAS2-1 plasmid and transformed into the yeast strain Y190. The β-galactosidase assay was performed for autoactivation test and a white colony was selected as the bait; 0.3 mg of DNA from a pACT2 *Arabidopsis* cell suspension cDNA library was transformed into the bait strain [32]. A total of 5 × 10^7^ transformants were selected first by β-galactosidase assay; blue colonies were further verified on synthetic dextrose medium lacking Leu, Trp, and His and containing 50 mM 3-aminotriazole. Well-grown colonies were taken as positive clones.

For validation of protein–protein interaction, the coding sequence of *HLH4* was cloned into the pGBKT7, and coding sequences of prey proteins (including CIB5, PRE1 and so on) were cloned into the pGADT7 vector. The resulting pGBKT7-bait and pGADT7-prey plasmids were co-transfected into the yeast strain Y2HGold and verified on selective media following a protocol of the Matchmaker Gold Yeast Two-Hybrid System (630489, Takara, Kyoto, Japan).

### 2.10. Bimolecular Fluorescence Complementation (BiFC) Assay

The BiFC assay was performed as described previously [33] with minor modifications. The full-length HLH4 coding sequence was fused with the N-terminal coding sequence of YFP (1–155 aa) and 3× Flag coding sequence and cloned into the pUC19 plasmid. Full-length coding sequences of *CIB5* and *PRE1* were fused with the C-terminal coding sequence of YFP (156–239 aa) and 3× Myc coding sequence and cloned into the pUC19 plasmid. Plasmids were co-transformed into *A. thaliana* protoplasts. GHD7-mCherry plasmid was used as a nuclear marker. YFP and mCherry fluorescence was imaged using a confocal microscope (TCS-SP8, Leica).

### 2.11. Protein Mass Spectrometry Analysis

Total protein of the HLH4 overexpression line 7 (OEHLH4-7) transgenic *A. thaliana* plants was extracted with protein extraction buffer (50 mM Tris-HCl (pH 7.5), 0.15 M NaCl, 5 mM MgCl_2_, 0.2% NP-40, 10% glycerol, and 1× protease inhibitor (Roche)). FLAG M2 magnetic beads (Sigma, M8823) 50 μL were added to the samples and incubated for 3 h with rotation at 4 °C. The beads were washed three times (5 min each) with protein extraction buffer and were transferred into a new tube in the last wash. The proteins were eluted from beads, separated via an SDS-PAGE, and used for mass spectrometry analysis.

### 2.12. Co-Immunoprecipitation (Co-IP) Assay

The plasmids used for the BiFC assay were co-transformed into *A. thaliana* protoplasts and incubated at 22 °C for 12–16 h in the dark. Protoplast total protein was extracted and added to 30 μL anti-c-Myc affinity gel (Sigma, E6654) for 4 h at 4 °C with rotation. The gel was washed three times with TTBS buffer (17 mM Tris, 130 mM NaCl, pH7.5, 1% Triton-X100) and boiled in 100 μL SDS protein sample buffer. The immunoprecipitated proteins were separated by an SDS-PAGE and transferred to a PVDF membrane. Anti-FLAG antibody (Sigma) and anti-c-Myc (Santa Cruz, 9E10) were used to detect the immunoprecipitated proteins, including HLH4-nYFP-3× FLAG, PRE1-nYFP-3×FLAG, CIB5-cYFP-3× Myc, and PRE1-cYFP-3× Myc.

### 2.13. RNA Sequencing

Leaves of five-week-old wild-type and HLH4 overexpression line 23 (OEHLH4-23) transgenic *A. thaliana* plants were collected for RNA isolation. rRNA-depleted RNA was used for RNA-seq library construction. RNA sequencing was performed on an Illumina Hiseq 4000 platform. Clean reads were mapped to the *A. thaliana* TAIR10 genome using PBS-GEM with default parameters [34]. StringTie was used for quantifying gene abundances [35] and the GFF3 annotation file (https://www.arabidopsis.org/, accessed on 21 March 2022) was used for identifying annotated reference genes. Gene expression levels were analyzed and normalized into RPKM (reads per kb per million mappedreads) values based on annotated *A. thaliana* gene models using DESeq2, and differentially-expressed genes (DEGs) between WT and HLH4OE-23 were identified with expression level fold changes > 2 [36]. DEGs in HLH4OE-23 were used for GO enrichment analysis using the online tool agriGOv2 (http://systemsbiology.cau.edu.cn/agriGOv2/ accessed on 2 December 2021) [37].

## 3. Results

### 3.1. Overexpression of HLH4 Leads to Plants with Dwarf and Dark Green Phenotypes

**Figure 1:**
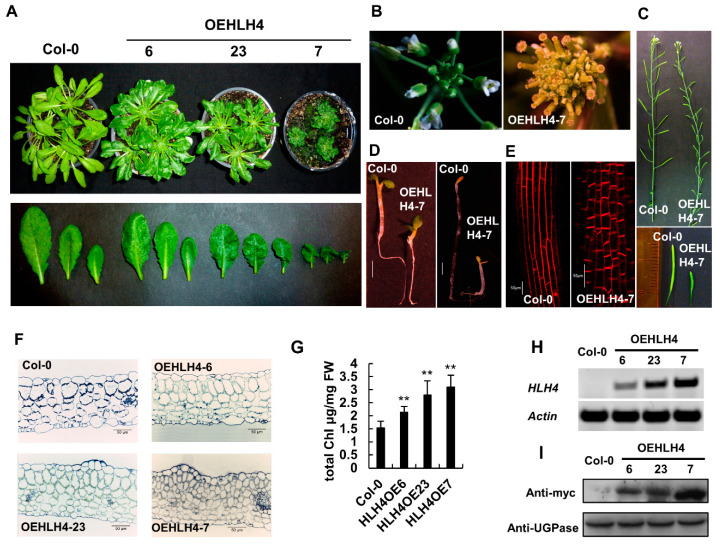
Overexpression of HLH4 causes dwarf and dark green phenotypes. (**A**) Phenotypes of whole plants and leaves of different HLH4 overexpression *A. thaliana* lines. (**B**) HLH4 overexpression lines showed abnormal flowers at the initial stage of flowering. (**C**) Siliques of HLH4 overexpression lines are shorter than those of wild-type plants. (**D**) Hypocotyls of HLH4 overexpression lines are shorter than wild-type (Col-0) plants grown under light or dark conditions, and cotyledons of HLH4 overexpression lines are unfolded in the dark. Bar = 1 mm. (**E**) Hypocotyl cells of HLH4 overexpression lines are shorter than those of wild-type plants. Seedlings were stained with propidium iodide and examined by confocal microscopy. Bar = 50 µm. (**F**) Leaf cross-sections of wild-type and different HLH4 overexpression lines. Bar = 50 µm. (**G**) Chlorophyll content of wild-type and different HLH4 overexpression lines. Data represent mean ± SD of three biological replicates with three technical replications (*n* = 9). Asterisks indicate significant differences (** *p* < 0.01). (**H**) Transcription levels of *HLH4* in leaves of wild-type and different HLH4 overexpression lines. (**I**) protein expression levels of different HLH4 overexpression lines.

Investigating transcription factors that regulate stress-dependent expression of the aldehyde dehydrogenase gene *ALDH7B4* in *A. thaliana* identified several transcription factors, including HLH4, which is annotated as an uncharacterized protein in TAIR [31]. When *HLH4* was overexpressed, around 50% of the overexpression lines (21 out of 38 transgenic lines) displayed dwarf and dark green phenotypes with varying intensities (Figure 1A). Several HLH4 overexpression lines showed abnormal flowers at the initial stage of flowering, with retarded elongation of stamens, petals and sepals leaving the pistils to stick out of the flower (Figure 1B). However, the reproductive organs of these lines became normal at later growth stages and generated shorter siliques with fertile seeds (Figure 1C). The hypocotyl of the *HLH4* overexpression lines is shorter than wild-type whether grown under light or dark conditions, and the cotyledons are unfolded in darkness (Figure 1D). Microscopic examinations of hypocotyls and leaf sections showed that while *HLH4* overexpression lines have the same mesophyll cell organization, cells are smaller and more compact than the wild-type (Figure 1E,F). This indicates that the dwarf phenotype of the *HLH4* overexpression lines is due to reduced cell elongation. Because of the higher density of mesophyll cells, the *HLH4* overexpression lines contain more chlorophyll than wild-type (Figure 1G), consistent with their dark green appearance. The severity of dwarfism is correlated with the expression level of *HLH4* detected both at the transcriptional (Figure 1H) and translational level (Figure 1I). These results indicate that overexpression of *HLH4* affects photomorphogenesis, inhibits cell elongation of various organs, and results in dwarf and dark green phenotypes.

### 3.2. HLH4 Is Localized in the Nucleus and Predominantly Expressed in Seedlings and Reproductive Organs

**Figure 2:**
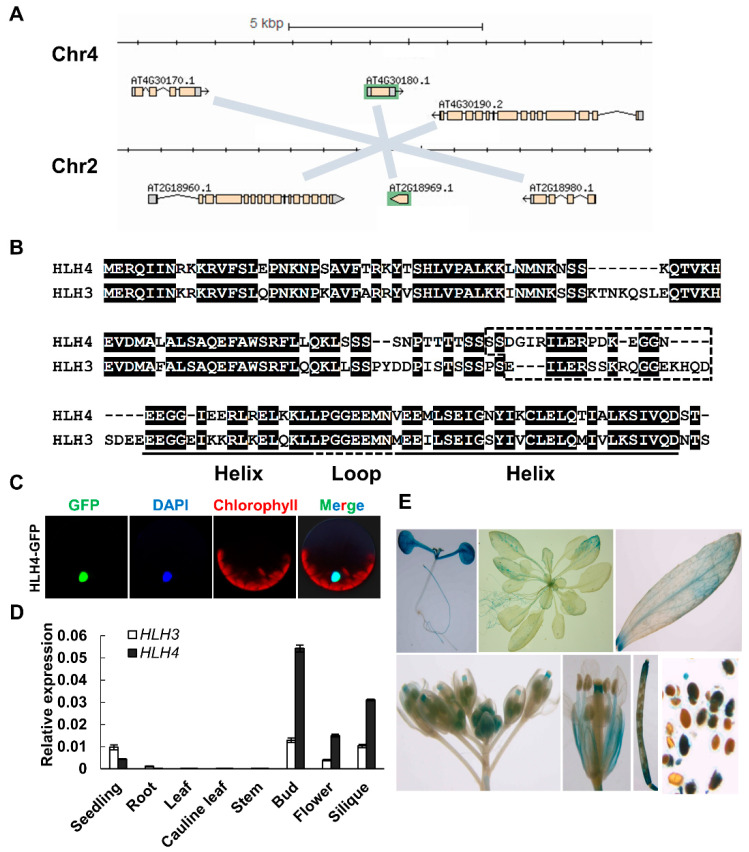
HLH4 is localized in the nucleus and is predominantly expressed in seedlings and reproductive organs. (**A**) Genome organization of HLH4 and synteny with its paralog HLH3 on *A. thaliana* chromosomes. (**B**) Protein sequence alignment of HLH4 and HLH3. Helix-loop-helix structure domains (https://www.predictprotein.org/, accessed on 21 March 2022) are indicated, and the dashed box represents hypothetical basic regions. (**C**) Subcellular localization of HLH4-GFP fusion protein in *A. thaliana* protoplasts. (**D**) RT-qPCR analysis of *HLH4* expression from various tissues. Data represent mean ± SD of three biological replicates with three technical replications (*n* = 9). (**E**) In vivo analysis of the HLH4 expression pattern using *proHLH4::GUS* reporter lines.

The *HLH4* gene has a paralog, *HLH3* (*At2g18969*). The *HLH4* and *HLH3* genes show synteny in the *A. thaliana* genome, suggesting that these two loci are segmental duplications (Figure 2A). *HLH4* and *HLH3* encode proteins with 158 and 175 amino acid residues, respectively. Helix structures are predicted in the C-terminal regions of the two proteins (Figure 2B). Amino acids (~17 aa) in front of the helix region of bHLH proteins comprise the basic region determining DNA binding activity. Although sequences of HLH4 and HLH3 are highly conserved, *HLH3* was excluded from the bHLH family in early reviews [38,39,40], and only later identified as a novel atypical bHLH protein [41]. *hlh4* and *hlh3* single mutants and *hlh4hlh3* double knock-out mutants did not show any phenotypic differences compared to wild-type plants under normal conditions (Figure S1).

While overexpression of HLH4 causes dwarf and dark green phenotypes, no phenotypic abnormalities were observed in *HLH3* overexpression lines (54 independent transgenic lines were examined), suggesting divergence of HLH4 and HLH3. The sequence of the basic region of HLH4 is not conserved with other bHLH family proteins and lacks the Glu-13/Arg-17, which is required for the E-box (5′-CANNTG-3′) DNA motif binding. Therefore, it was predicted as a HLH protein without DNA binding ability [39]. To verify the DNA binding ability of HLH4, a 39 bp DNA fragment of the *A. thaliana ALDH7B4* promoter harboring the conserved G-box (5′-CACGTG-3′) was used as a probe in an EMSA assay. The results showed that HLH4 did not bind to the G-box containing DNA fragment (Figure S2). A nuclear localization signal (NLS) “RKKR” was predicted in the N-terminal part of HLH4 [42], and the nucleus localization was confirmed by transient expression of the *HLH4-GFP* fusion gene in protoplasts (Figure 2C). RT-qPCR analysis showed that HLH4 is mainly expressed in seedlings and reproductive organs, which is in line with the GUS reporter-driven analysis of the *HLH4* promoter (Figure 2D,E). In summary, HLH4 is a nucleus-localized transcription factor without G-box DNA binding ability, and it is predominantly expressed in seedlings and reproductive organs.

### 3.3. HLH4 Negatively Regulates Cell Elongation and Anthocyanin Biosynthesis-Associated Genes

**Figure 3:**
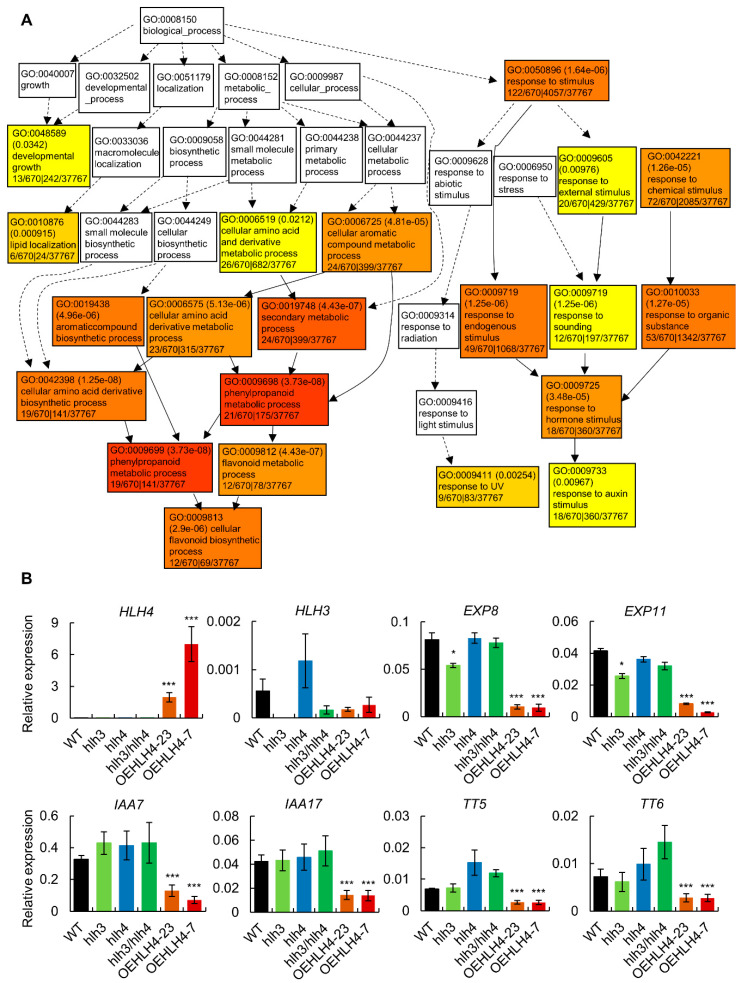
Transcriptomic analyses of RNA-seq Data and RT-qPCR validation. (**A**) Gene ontology enrichment analysis of down-regulated DEGs (differentially expressed genes) in O23 leaves. (**B**) RT-qPCR analysis of selected DEGs. Data represent mean ± SD of three biological replicates with three technical replicates (*n* = 9). Asterisks indicate significant differences (* *p* < 0.05, *** *p* < 0.001).

To investigate the transcriptional regulation governed by HLH4, we compared the transcriptome of the *HLH4* overexpression line O23 (OEHLH4-23) and wild-type plants grown under normal conditions. Compared to wild-type, a total of 697 and 509 genes were downregulated and upregulated (|log2 fold change| > 2) in O23, respectively (Table S1). To investigate the possible biological processes that HLH4 involves, the differentially-expressed genes were used for gene ontology analysis. The results showed that the downregulated genes are significantly enriched in developmental growth, flavonoid biosynthesis, response to UV, and auxin stimulus (Figure 3A), suggesting HLH4 may be involved in the regulation of genes that function in these biological processes.

**Figure 4:**
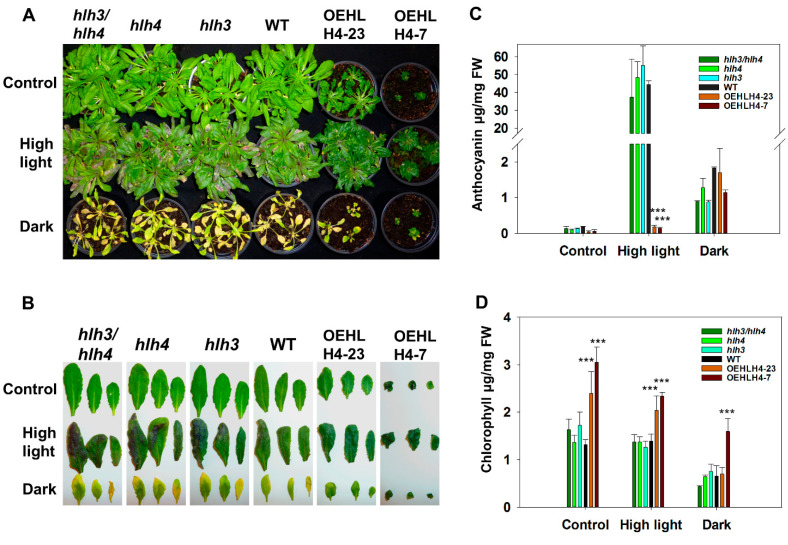
Overexpression of HLH4 prevents anthocyanin accumulation. (**A**) Four-week-old HLH4 overexpression, *hlh4*, *hlh3*, *hlh3hlh4*, and wild type *A. thaliana* plants were treated with darkness or high light stress for seven days. (**B**) Leaf samples from plants as shown in (**A**). (**C**) Chlorophyll contents of plants as shown in (**A**). (**D**) Anthocyanin contents of plants as shown in (**A**). Data represent mean ± SD of three biological replicates with three technical replications (*n* = 9). Asterisks indicate significant differences (*** *p* < 0.001).

To confirm the data from the RNA-seq analysis, we detected the expression of *HLH4* and *HLH3* and selected several genes, including *EXP8* and *EXP11* (which are involved in cell elongation), *IAA7* and *IAA17* (involved in auxin and BR signaling), and *TT5*, *TT6*, *MYB75*, and *TT3* (which are involved in flavonoid biosynthesis) for validation using RT-qPCR and RT-PCR (Figure 3B and Figure S3). *HLH4* was highly expressed in OEHLH4-23 and OEHLH4-7 compared with WT and *hlh3*, *hlh4* and *hlh3hlh4*, confirming the materials we used for RNA-seq. *HLH3* seems slightly upregulated in *hlh4*, probably because the loss of *HLH4* transcripts requires activated *HLH3* to compensate for their function. Consistent with the RNA-seq data, all the genes were significantly downregulated in different *HLH4* overexpression lines (Figure 3B and Figure S3), while *DECREASE WAX BIOSYNTHESIS* (*Dewax*) and *KELCH REPEAT F-BOX* (*KFB20*) were upregulated. However, expression of these genes was not significantly changed in *hlh3*, *hlh4* and *hlh3hlh4* mutants (Figure 3B). Because many downregulated genes in *HLH4* overexpression lines are involved in flavonoid biosynthesis and response to UV, we suppose that *HLH4* may negatively regulate flavonoid biosynthesis. Light stress induces the accumulation of anthocyanin, a type of flavonoid, to protect the photosynthetic apparatus against excess light [43]. Therefore, plants were treated with high-light or dark conditions to analyse the influence of overexpression of *HLH4* in anthocyanin accumulation. However, no significant differences in anthocyanin accumulation were observed among wild-type, *HLH4* overexpression lines, *hlh3*, *hlh4*, and *hlh3hlh4* mutants under normal and dark conditions (Figure 4A). Light stress strongly induced anthocyanin accumulation in wild-type, *hlh3*, *hlh4*, and *hlh3hlh4* plants; however, in *HLH4* overexpression lines, anthocyanins did not accumulate (Figure 4C). Anthocyanin content has a negative relation with chlorophyll in colorful tree species [44]. Therefore, the chlorophyll content was determined in treated *A. thaliana* plants. Consistent with the observations in colorful tree species, *HLH4* overexpression lines contain more chlorophyll than wild-type, *hlh3*, *hlh4*, and *hlh3hlh4* plants under all tested conditions (Figure 4D).

### 3.4. HLH4 Interacts with CIB1 and PRE1

Because HLH4 represses gene transcription without DNA binding ability, we assumed that HLH4 executes the repression through interacting with other transcription factors. We therefore performed a yeast two-hybrid (Y2H) screen to identify proteins that interact with HLH4. The screening identified 22 proteins from 27 positive clones (Figure S4, Table S2), including three bHLH proteins (bHLH49, bHLH69, bHLH76) which were previously predicted to bind to an E-box [39]. According to a phylogenetic analysis, both bHLH76 and bHLH49 belong to subfamily eighteen. bHLH76 and bHLH49, known as CIB5 (CRY2-interacting bHLH 5) and CIL1 (CIB1 LIKE PROTEIN 1), respectively, are involved in floral initiation by forming a complex with cryptochrome 2 (CRY2) [45]. bHLH69, known as LRL2, belongs to subfamily seventeen and is involved in the modulation of circadian rhythm [46] and root hair development [47]. It has been reported that CIB5 and CIL1 have the highest binding affinity to a G-box both in vitro and in vivo [48]. Among the proteins identified in the yeast two-hybrid screen are protein modifiers, including homoserine kinase, serine acetyltransferase1, and F-box/kelch-repeat protein SKIP30, indicating that these proteins might modify HLH4. Several proteins related to the GTPase, transport, and hormone pathways were identified as well, suggesting the putative involvement of HLH4 in these pathways. In addition, there were six uncharacterized proteins showing interaction with HLH4, suggesting that HLH4 together with these proteins might be involved in pathways that remain unknown.

In addition to the Y2H screening, we analyzed the HLH4 interactome by affinity purification of FLAG-tagged HLH4 from total proteins of *35S-HLH4-3 ×* *FLAG-3 × MYC* transgenic *Arabidopsis* plants. The co-purified proteins were subjected to mass spectrometry analysis (IP_Mass). Among the co-purified proteins, there were three PRE transcription factors, *AT5G39860*, *AT1G26945*, and *AT5G15160*, belonging to an atypical non-DNA binding bHLH family (Table S3).

**Figure 5:**
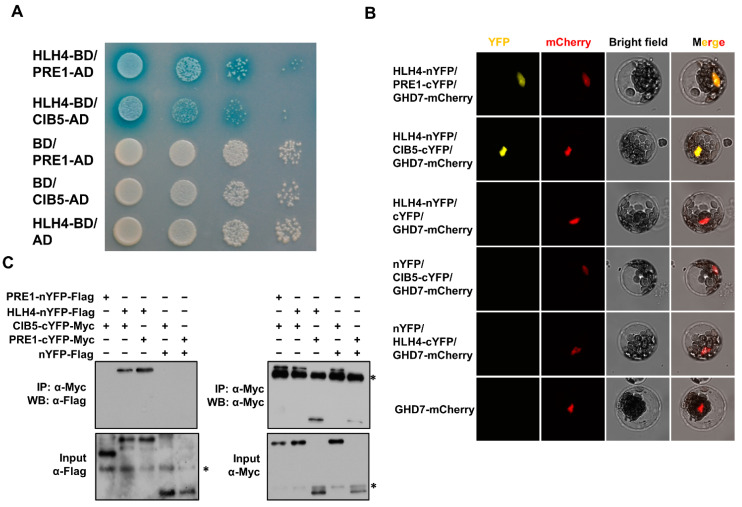
HLH4 interacts with CIB1 and PRE1. Yeast two hybrid (**A**), BiFC (**B**) and protoplast co-immunoprecipitation (Co-IP) (**C**) assays indicate that HLH4 interacts with CIB1 and PRE1. The GHD7-mCherry plasmid was used as a nuclear marker. Asterisk * in (**C**) represents unspecific protein bands.

We further selected CIB5 and PRE1 as representatives to confirm their interaction with HLH4 by Y2H and co-immunoprecipitation (co-IP) as well as bimolecular fluorescence complementation (BiFC) assays. HLH4-BD/PRE1-AD and HLH4-BD/CIB5-AD yeast transformants turned blue on SD-trp-leu medium in a β-galactosidase assay (Figure 5A). A YFP signal was detected in protoplasts co-transformed with HLH4-YFPN/PRE1-YFPC or HLH4-YFPN/CIB5-YFPC, and not detected with other tested YFP fused protein combinations (Figure 5B), supporting the in vivo interaction of HLH4 with PRE1 and CIB5. Protoplast co-immunoprecipitation analysis showed that all transformed plasmids were expressed, as the proteins in input samples were detected by Western blot. HLH4 was co-immunoprecipitated with PRE1 and CIB5, confirming the interactions of HLH4 with PRE1 and CIB5 (Figure 5C). However, PRE1 cannot be co-immunoprecipitated with CIB5, indicating that they do not directly interact with each other (Figure 5C).

As Y2H screening identified two CIB proteins and IP_Mass identified three PRE proteins, Y2H β-galactosidase assays were performed to check whether HLH4 interacts with all CIB and PRE proteins. HLH4 interacts with all six PRE proteins and seven CIB proteins, including CIL1 and CIL2 (Figure S5). We further found that while HLH4 interacts with other members of subfamily seventeen and eighteen bHLHs, including BEE and LRL proteins, it does not interact with ICE1 and PIF4, which belong to subfamily nine and fifteen, respectively [39]. In addition, HLH4 interacts with itself to form homodimers and its paralog HLH3 (Figure S5). These results indicate that HLH4 interacts with subfamily seventeen and eighteen bHLH proteins, which are typical bHLH transcription factors and have DNA binding abilities, as well as with atypical bHLH transcription factors, including PRE proteins and itself.

### 3.5. HLH4 Forms a Triantagonistic System with CIB5 and PRE1 to Repress Target Gene Expression

**Figure 6:**
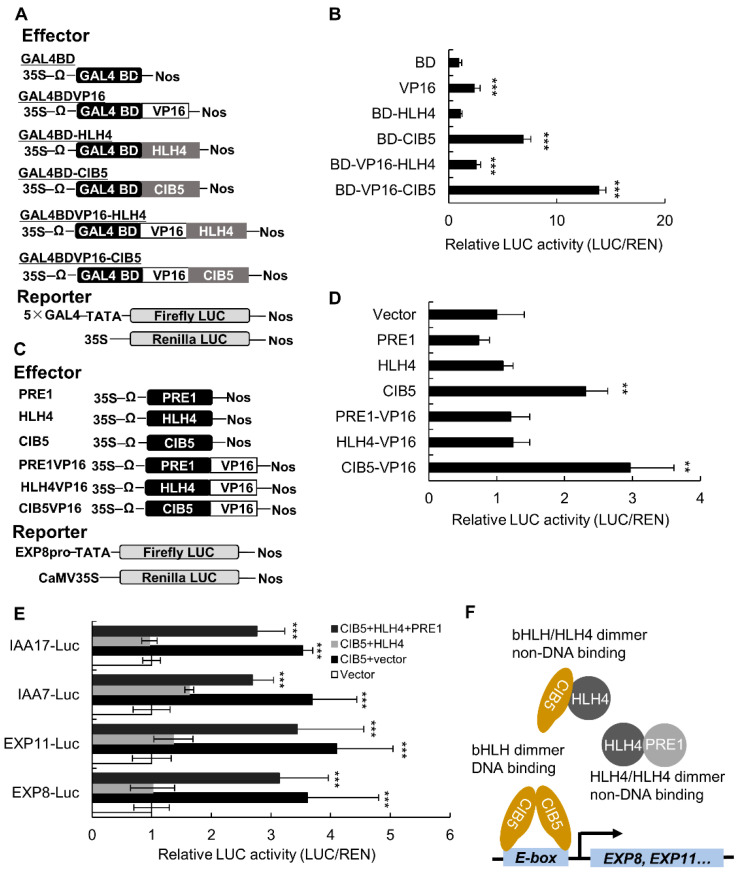
HLH4 forms a triantagonistic complex with CIB5 and PRE1 to repress target gene expression. (**A**) Schematic representation of the constructs used in the transient expression assay. The pro35S-GAL4-LUC reporter construct (5×Gal4-LUC) harbors a firefly luciferase (LUC) coding sequence driven by the CaMV 35S promoter with five copies of the GAL4-responsive element;proAtUbiquitin3:REN (Renilla LUC) served as an internal control. HLH4, PRE1 and CIB5 coding sequences fused to the GAL4 DNA binding domain (BD) or VP16 activation domain driven by the CaMV 35S promoter were used as effector constructs. (**B**) Relative luciferase activities detected in *A. thaliana* protoplasts transformed with the plasmids shown in (**A**). The luciferase activity of the empty vector with the BD coding sequence was set as 1. (**C**) Schematic representation of the constructs used in transient expression analysis; *proEXP8-LUC* reporter gene contains a LUC coding sequence driven by 2 kb *EXP8* promoter and the effector constructs contain the protein-coding region of HLH4, PRE1 and CIB5 or fused with VP16 activation domain driven by the CaMV 35S promoter. (**D**) Relative luciferase activities detected in *A. thaliana* protoplasts transformed with the plasmids shown in (**C**). (**E**) Relative luciferase activities detected in *A. thaliana* protoplasts co-transformed with the plasmids shown in (**A**). The LUC reporter was driven by *EXP8*, *EXP11*, *IAA7*, and *IAA17* promoters. (**F**) A model of a triantagonistic bHLH system composed of HLH4, CIB5 and PRE1 which regulates cell elongation in *A. thaliana*. CIB5 homodimers can bind to the promoter and activate the expression of genes related to cell elongation. HLH4 forms heterodimers with CIB5 and interferes with the DNA binding ability of CIB5, thereby inhibiting target gene transcription. PRE1 forms heterodimers with HLH4 and suppresses the inhibitory activity of HLH4 on CIB5. Data represent mean ± SD of three biological replicates with three technical replications (*n* = 9). Asterisks indicate significant differences (** *p* < 0.01, *** *p* < 0.001).

Because HLH4 does not bind the G-box DNA motif and is probably without DNA binding ability, we examined whether HLH4 functions as a repressor. Transient expression of HLH4 fused with the yeast GAL4 DNA binding domain (BD-HLH4) in *A. thaliana* protoplasts did not activate or repress a GAL4-LUC reporter gene containing a 5× GAL4 binding motif in its promoter (Figure 6A,B). Consistently, transient expression of HLH4 fused with the BD and VP16 activator domain (residues 413–490 of the herpesvirus protein VP16) did not suppress the transcriptional activation activity of VP16. These results indicate that HLH4 does not have a direct repressor activity and probably acts as an indirect repressor. On the contrary, CIB5 significantly activated the GAL4-LUC reporter gene when fused with BD and enhanced the transcriptional activation activity of VP16 when fused with BD and VP16, suggesting that CIB5 is a transcriptional activator.

We next performed transient expression assays in which a 2 kb *EXP8* promoter sequence containing thirteen E-box motifs was placed upstream of the *LUC* reporter gene and used as a reporter. *EXP8* was reported to be a downstream target gene of CIB5, and CIB5 specifically binds the G-box-like cis-elements within the *EXP8* promoter [20]. Our results show that the activity of the *EXP8-LUC* reporter gene was significantly upregulated when *pro35S-CIB5* or *pro35S-CIB5-VP16* were coexpressed with the *EXP8-LUC* reporter gene (Figure 6C,D),which confirms that CIB5 binds to the *EXP8* promoter. On the contrary, the reporter gene was not activated when *pro35S:HLH4*, *pro35S-HLH4-VP16*, *pro35S-PRE1* and *pro35S-PRE1-VP16* were coexpressed with the *EXP8-LUC* reporter, indicating that HLH4 and PRE1 do not bind to DNA.

As HLH4 interacts with PRE1 and CIB5 and CIB5 has transcriptional activation activity, we next analyzed whether HLH4 interferes with the activity of CIB5. When the *proEXP8-LUC* reporter was co-transformed with *pro35S-HLH4* and *pro35S-CIB5* effectors the activation activity of CIB5 on the *proEXP8-LUC* reporter gene was decreased, indicating HLH4 suppresses CIB5 (Figure 6E). Moreover, the suppression of the HLH4 effector was alleviated and activity of the *proEXP8-LUC* reporter was restored when the *pro35S-PRE1* effector was coexpressed with *pro35S-HLH4* and *pro35S-CIB5* effectors (Figure 6E). Similar transcription activation patterns were observed for the *proEXP11-LUC*, *proIAA7-LUC*, and *proIAA17-LUC* reporter genes (Figure 6E, Figure S6A). However, *pro35S-CIB5* could not activate the *proTT5-LUC*, *proTT6-LUC*, *proTT8-LUC*, and *proMYB75-LUC* reporter genes (Figure S6). These results indicate that the expression of the cell elongation-related genes *IAA7*, *IAA17*, *EXP8*, and *EXP11* could be regulated by a triple bHLH complex involving a bHLH activator, namely CIB5, and two atypical HLH modulators, HLH4 and PRE1. By interacting with CIB5, HLH4 interferes with the activity of CIB5, thus inhibiting the transcription of target genes regulated by CIB5 (including *EXP8*, *EXP11*, *IAA7*, and *IAA17*). The interference of HLH4 on CIB5 is counteracted by PRE1, in which these bHLH proteins form a new tri-antagonistic complex (Figure 6F).

## 4. Discussion

In this study, we showed that overexpression of the uncharacterized bHLH transcription factor HLH4 leads to abnormal phenotypes, including dwarf varieties as well as ones with dark green leaves, short hypocotyl, and defective flowers. The HLH4 overexpression lines accumulate much less anthocyanin than wild-type *A. thaliana* plants in response to high light stress. Our study demonstrates that HLH4 interacts with other bHLH proteins that regulate plant cell elongation, including CIB5 and PRE1, to form a new triantagonistic complex. HLH4 and PRE1 antagonistically modulate the expression of downstream genes such as *EXPANSINS* through HLH4 interaction with CIB5.

bHLH proteins belong to one of the largest transcription factor families in plants, and are involved in a myriad of regulatory processes [41]. Typical bHLH factors bind to an E-box (CANNTG) DNA motif, predominantly the G-box (CACGTG) motif, through their basic domains, and can form homo- or heterodimers with the HLH domain [39]. About one-fourth of plant bHLH proteins contain only the HLH domain and lack a basic domain, known as HLH or atypical bHLH factors [41]. Atypical bHLH factors dimerize with typical bHLHs, forming heterodimers that cannot bind DNA, and thus disrupt the transcriptional activity of typical bHLH transcription factors [39,41]. However, PREs(which are atypical bHLH factors) activate typical bHLHs by interacting with atypical bHLH factors that interfere with the DNA binding ability of the typical bHLH proteins [12,13,14]. This negative regulation of the inhibitor of a typical active protein forms a triantagonistic module, and provides additional regulatory mechanisms to integrate multiple developmental and environmental signals. We provide evidence that HLH4 is without DNA binding ability. First, the basic domain residues of HLH4 are not conserved as in typical bHLH factor basic domains, and lack the residues required for DNA binding (Figure 2B). Second, HLH4 did not bind the DNA probe containing a G-box in vitro (Figure S2). Third, HLH4-VP16 cannot activate the *EXP8* promoter activity, indicating HLH4 cannot bind the *EXP8* promoter sequence, which contains many E-box motifs (Figure 6C). Therefore, HLH4 appears to execute regulatory functions through interaction with other DNA binding bHLH factors.

Several studies have demonstrated that the CIB and PRE family proteins promote environmental and hormonal-induced cell elongation [12,13,14,15,49,50]. CIB5 and its paralog CIB1 are typical bHLH proteins and bind to the E-box of target genes, including *EXP8* and *EXP11*, which encode cell wall loosening enzymes [19,20,46]. PRE family members indirectly promote cell elongation by suppressing atypical bHLH proteins, including IBH1 and AIFs, through interaction and forming heterodimers [12,14,15]. We found that HLH4 interacts with CIB5 and PRE1 to form a new triantagonistic complex in which HLH4 plays a similar role as in IBH1- and AIF-composed triantagonistic systems [20,51]. Thus, HLH4 was identified as a new player in the bHLH/HLH/HLH triantagonistic system regulating cell elongation.

Overexpression of many atypical bHLH factors such as AIF1, AIF2, AIF3 [51], PAR1 [52], and IBH1 [20,53] exhibited dwarfism and dark green leaves, which are similar to the phenotype of HLH4-overexpression plants, suggesting that these atypical bHLH factors may have similar biological functions in controlling cell elongation. This functional redundancy may explain why the loss-of-function mutants of these genes did not show clear phenotypes (Figure S1). However, HLH3-overexpression transgenic *A. thaliana* plants did not show any phenotypes, indicating that HLH3 functionally diverged from HLH4 even though the sequences of HLH3 and HLH4 are fairly conserved. Previous studies showed that brassinosteroid (BR) signaling upregulates and downregulates PRE1 and IBH1, respectively, due to BZR1 functions opposite transcriptional regulation activities at their promoters [15]. In response to BR, the cellular level of IBH1 would be effectively decreased by protein interaction with PRE1 and repression by BZR1 on the transcriptional level [15]. The relative HBI1 level would consequently be increased due to the decreased IBH1, although BR does not affect the HBI1 transcription [54]. IAA7 and IAA17, which are important players in auxin-signaling, are involved in brassinosteroid (BR) responses. Overexpression of HLH4 significantly decreased the transcript levels of *IAA7* and *IAA17* (Figure 3B), and *iaa7*, *iaa17* mutants showed aberrant BR sensitivity and similar dwarf phenotypes to HLH4 overexpression lines [55]. Together, these studies suggest that the dwarf phenotype of HLH4 overexpression lines may result in the disruption of BR regulated cell elongation.

Previous studies mainly focused on the dwarf phenotype induced by overexpression of atypical bHLH factors. Our RNA-seq analysis disclosed that overexpression of HLH4 results in the downregulation of many genes involved in the flavonoid biosynthetic process (Figure 3A), suggesting HLH4 is involved in the regulation of the flavonoid biosynthesis. Indeed, overexpression of HLH4 prevents anthocyanin accumulation in response to high light stress. Although genes like *TT5*, *TT6*, *TT8*, and *MYB75,* which are involved in flavonoid biosynthesis, were downregulated by the overexpression of HLH4, their expression was not regulated by CIB5 (Figure 6E). In addition to CIB5, we showed that HLH4 interacts with many other bHLH factors (Figure S5), suggesting HLH4 may be involved in the regulation of multiple biological pathways, including flavonoid biosynthesis and photomorphogenesis (Figure 1D), by forming other HLH/bHLH transcription regulatory complexes. Further dissection of the protein–DNA and protein–protein interactions of HLH/bHLH transcription networks will promote understanding of the regulatory mechanisms of plant growth and environmental responses.

## Data Availability

The datasets generated during and/or analysed during the current study are available from the corresponding author on a reasonable request.

## Supplementary Materials

The following supporting information can be downloaded at: https://www.mdpi.com/article/10.3390/cells11071087/s1, Figure S1: Phenotyping and genotyping the hlh4, hlh3, hlh3hlh4; Figure S2: EMSA showing HLH4 does not bind a G-box-containing ALDH7B4 promoter probe; Figure S3: Validation of DEGs identified from RNA-seq by RT-PCR (A) and RT-qPCR (B) analyses; Figure S4: Proteins identified as HLH4 interactors by Y2H library screening; Figure S5: Y2H to show HLH4 interacts with other bHLH proteins; Figure S6: Relative luciferase activities detected in A. thaliana protoplasts co-transformed with the plasmids shown in Figure 6A; Table S1. Differentially expressed genes from RNA-seq; Table S2. HLH4 interacting proteins identified by Y2H screening; Table S3. HLH4OE23-Flag Immunoprecipitation-Mass Spectrometry; Table S4: Primers used in this study.
