# Supplementary material for: Overexpression of HLH4 Inhibits Cell Elongation and Anthocyanin Biosynthesis in Arabidopsis thaliana
Source: Cells. 2022 Mar 24;11(7):1087. doi: 10.3390/cells11071087 (PMC8997993; doi:10.3390/cells11071087)
Supplement: Supplementary file 1 [file cells-11-01087-s001.zip › suppl/Supplenmental figures.pdf]

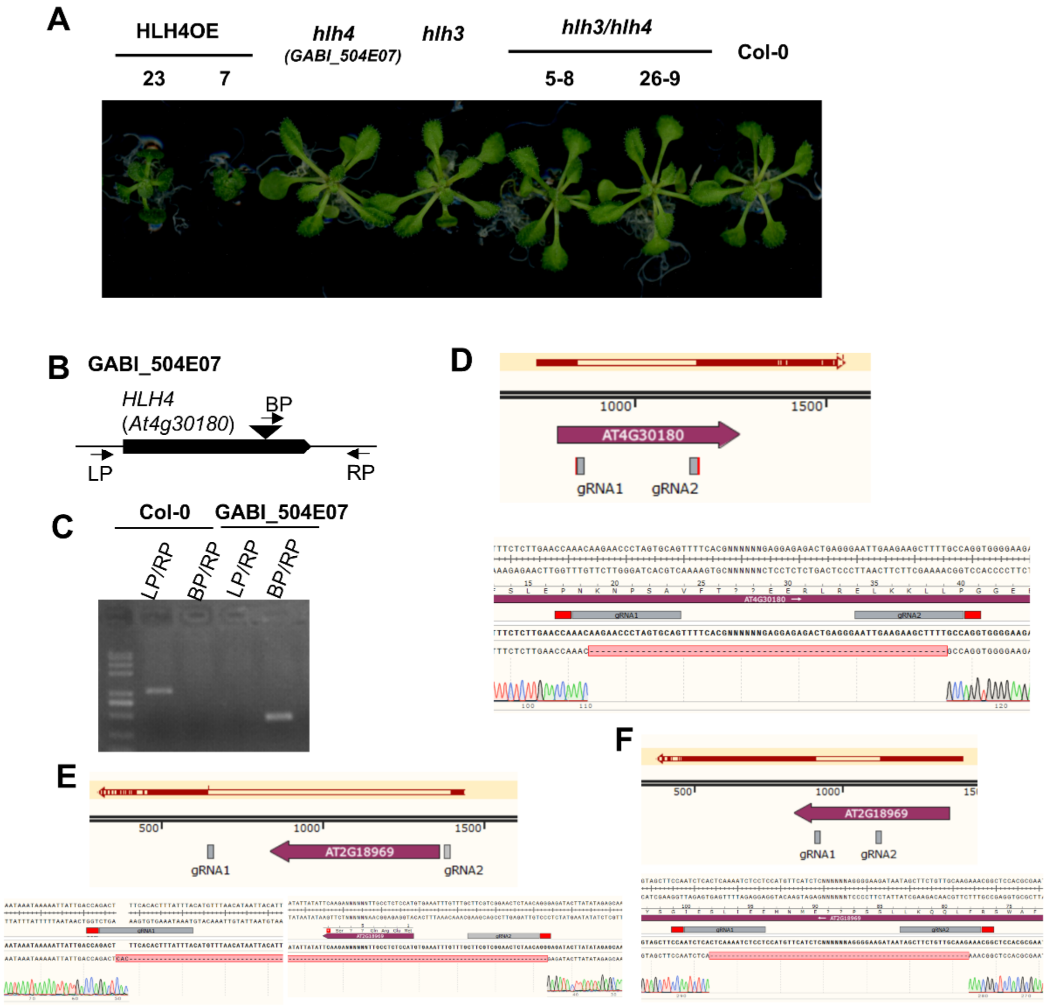

Figure S1. Phenotyping and genotyping the *hlh4*, *hlh3*, *hlh3hlh4*. (**A**) Phenotypes of 3-week HLH4 overexpression -23 and -7, *hlh4*, *hlh3*, *hlh3hlh4* and wildtype plants grown on ½ MS medium. (**B**) Schematic representation of the *hlh4* mutant (GABI_504E07). (**C**) Genotyping of the *hlh4* mutant. (**D**) Sequencing analysis of the *HLH4* locus of the *hlh4* mutant generated by CRISPR/Cas9. (**E**) Sequencing analysis of the *hlh3* mutant generated by CRISPR/Cas9. (**F**) Sequencing analysis of the *HLH3* locus of *hlh3hlh4* mutant generated by CRISPR/Cas9. This hlh3 mutation cross with *hlh4* mutant (GABI_504E07) results in the *hlh3hlh4* mutant (5-8) and cross with hlh4 mutant shown in D results in the *hlh3hlh4* mutant (26-9) .

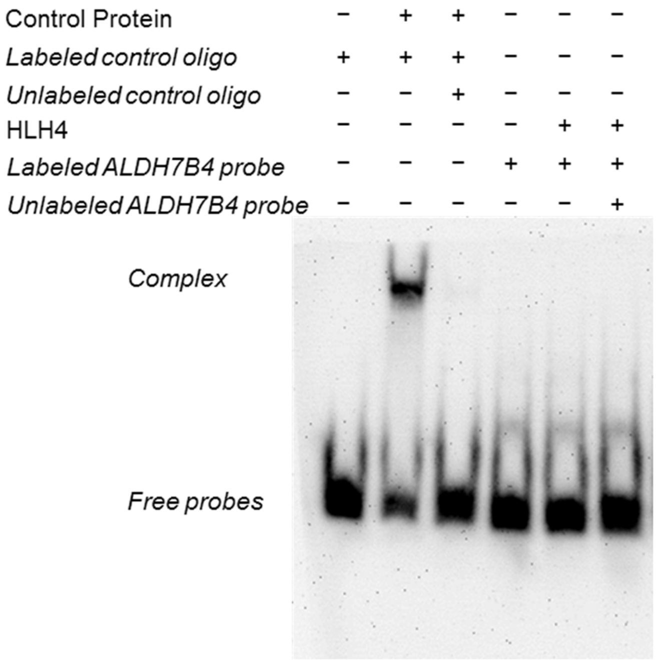

Figure S2. EMSA showing HLH4 dose not bind a G-box containing *ALDH7B4* promoter probe.

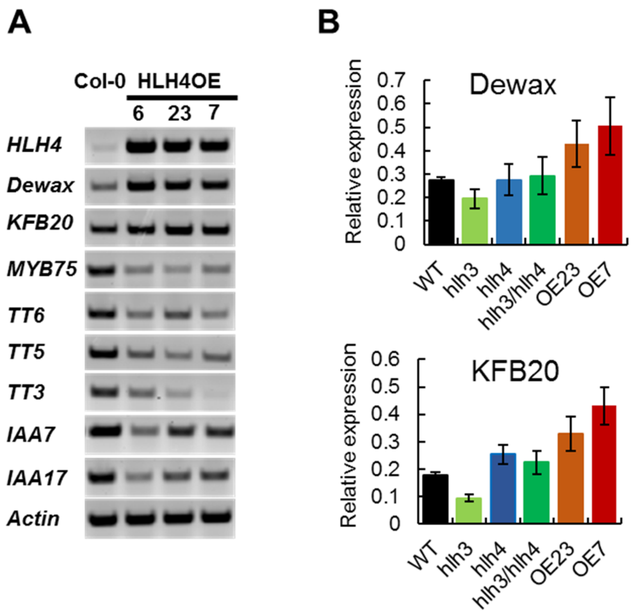

Figure S3. Validation of DEGs identified from RNA_seq by RT-PCR (A) and RT-qPCR (B) analyses.

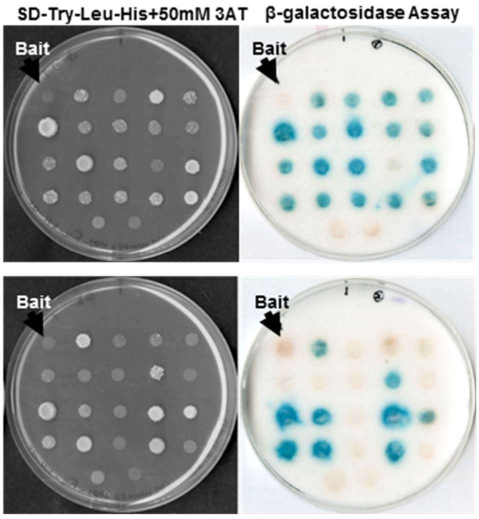

Name	Locus	Description
CIB5	AT1G26260	transcription factor bHLH76
CIL1	AT1G68920	transcription factor bHLH49
LRL2	AT4G30980	transcription factor bHLH69
RPL23A	AT1G04480	60S ribosomal protein L23
RPS15	AT1G04270	40S ribosomal protein S15-1
HSK	AT2G17265	homoserine kinase
SAT5	AT5G56760	serine acetyltransferase 1
SKIP30	AT3G63220	F-box/kelch-repeat protein
RAN2	AT5G20020	GTP-binding nuclear protein
TCTP1	AT3G16640	translationally-controlled tumor protein
GDI1	AT2G44100	guanosine nucleotide diphosphate dissociation inhibitor 1
ENODL19	AT4G12880	early nodulin-like protein 19
DIT1	AT5G12860	dicarboxylate transporter 1
ACO2	AT1G62380	1-aminocyclopropane-1-carboxylate oxidase 2
GASA4	AT5G15230	gibberellin-regulated protein 4
PGIP1	AT5G06860	polygalacturonase inhibitor 1
AT1G51020	AT1G51020	uncharacterized protein
AT5G06590	AT5G06590	uncharacterized protein
AT5G39570	AT5G39570	uncharacterized protein
AT5G47060	AT5G47060	uncharacterized protein
AT3G01430	AT3G01430	uncharacterized protein
AT5G14410	AT5G14410	uncharacterized protein

Figure S4. Proteins identified as HLH4 interactors by Y2H library screening.

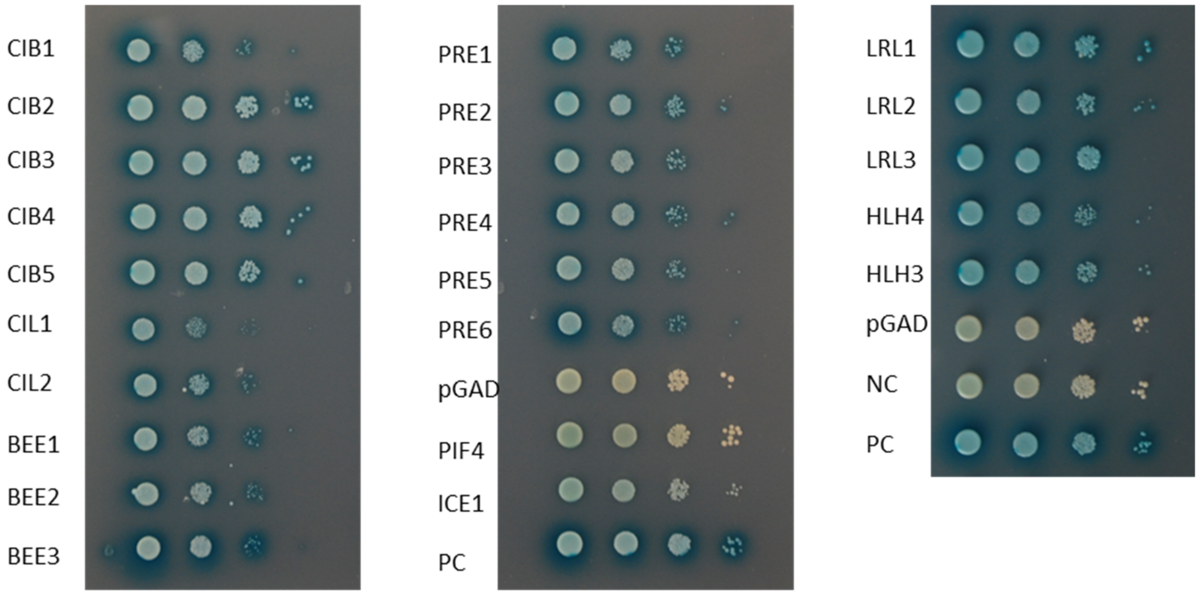

Figure S5. Y2H to show HLH4 interacts with other bHLH proteins. HLH4 coding sequence was fused with GAL4 BD as bait, while other bHLH proteins were fused with GAL4 AD as preys.

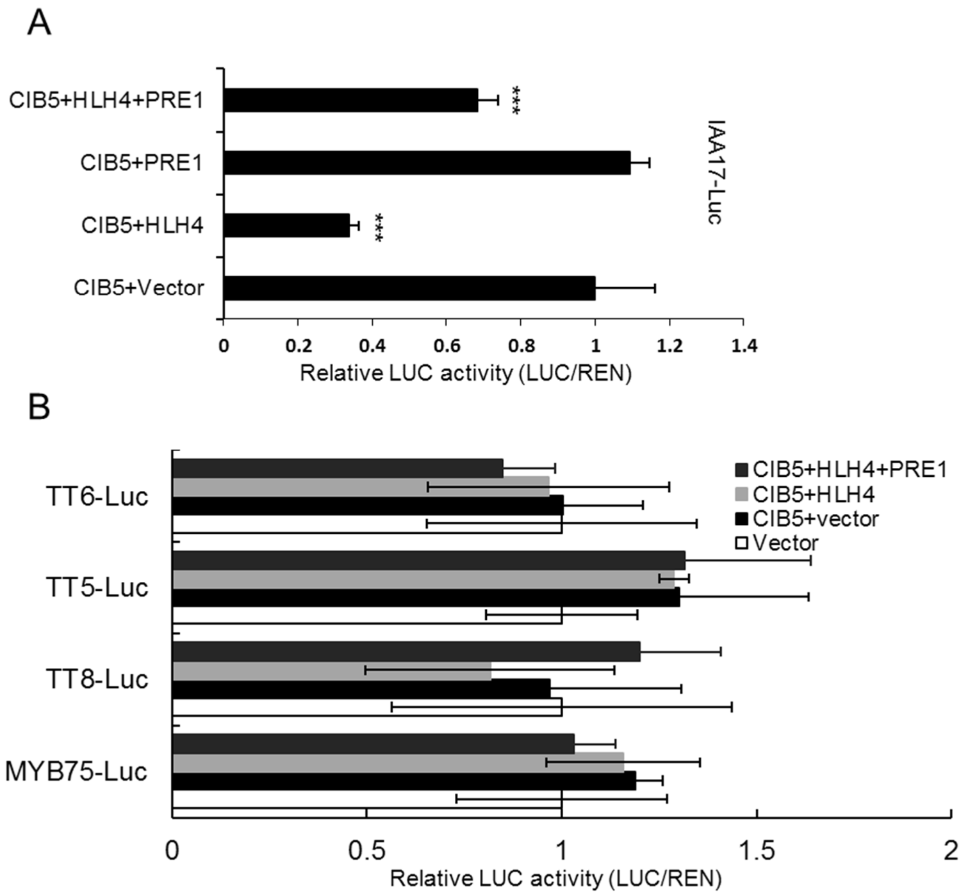

Figure S6. Relative luciferase activities detected in *A.thaliana* protoplasts co-transformed with the plasmids shown in Figure 6A. (A) The LUC reporter was driven by the *IAA17* promoter. (B) The LUC reporter was driven by *TT5*, *TT6*, *TT8* and *MYB75* promoters. Data represent mean ± SD of three biological replicates with three technical replicates (n = 9). Asterisks indicate significant differences (*** $p < 0.001$).
